# Design and In Vitro Evaluation of Cross-Linked Poly(HEMA)-Pectin Nano-Composites for Targeted Delivery of Potassium Channel Blockers in Cancer Therapy

**DOI:** 10.3390/gels12010013

**Published:** 2025-12-24

**Authors:** Gizem Ozkurnaz Civir, Fatemeh Bahadori, Ozgur Ozay, Gamze Ergin Kızılçay, Seyma Atesoglu, Ebru Haciosmanoglu Aldogan, Burak Celik

**Affiliations:** 1 Department of Biotechnology, Institute of Health Sciences, Bezmialem Vakif University, 34093 Fatih, Istanbul, Türkiye; gizemozkurnaz@comu.edu.tr (G.O.C.); seybulut@gmail.com (S.A.); 2 Department of Analytical Chemistry, Faculty of Pharmacy, Istanbul University-Cerrahpasa, Buyukcekmece Campus, 34500 Buyukcekmece, Istanbul, Türkiye; 3 Department of Pharmaceutical Biotechnology, Faculty of Pharmacy, Bezmialem Vakif University, 34093 Fatih, Istanbul, Türkiye; eczburakcelik@gmail.com; 4 Laboratory of Biomaterials Research, Department of Bioengineering, Faculty of Engineering, Çanakkale Onsekiz Mart University, 17100 Çanakkale, Türkiye; 5 Department of Analytical Chemistry, Faculty of Pharmacy, Istanbul University, Beyazıt, 34116 Fatih, Istanbul, Türkiye; gamze.erginkizilcay@istanbul.edu.tr; 6 Department of Medical Biology, Faculty of Medicine, Bezmialem Vakif University, 34093 Fatih, Istanbul, Türkiye; 7 Department of Biophysics, Faculty of Medicine, Bezmialem Vakif University, 34093 Fatih, Istanbul, Türkiye; ebru.aldogan@iuc.edu.tr; 8 Department of Biophysics, Cerrahpasa Faculty of Medicine, Istanbul University-Cerrahpasa, 34500 Fatih, Istanbul, Türkiye

**Keywords:** HEMA-pectin nanoparticles, potassium channel blockers, dofetilide, azimilide, targeted cancer therapy, EPR effect, A549 lung cancer cells

## Abstract

Potassium (K^+^) channel blockers are promising anticancer agents but suffer from off-target toxicities. We designed cross-linked poly-2-Hydroxyethyl methacrylate (HEMA)–pectin nanogels (HPN) to deliver two model blockers—dofetilide (Dof) and azimilide (Azi)—and evaluated their physicochemical properties, release behavior, and in vitro anticancer activity. HPN was synthesized by surfactant-assisted aqueous nanogel polymerization and comprehensively characterized (FTIR, DLS, TEM/SEM, XRD, BET). The particles were monodispersed with a mean diameter ~230 nm, compatible with tumor accumulation via the Enhanced Permeability and Retention (EPR) effect, and exhibited a microporous matrix suitable for controlled release. Drug loading was higher for Dof than for Azi, with DL% values of 82.30 ± 3.1% and 17.84 ± 2.9%, respectively. Release kinetics diverged: Azi-HPN followed primarily first-order diffusion with a rapid burst, whereas Dof-HPN showed mixed zero/first-order behavior. Cytotoxicity was assessed in A549 lung cancer and BEAS-2B bronchial epithelial cells. Both free and nano-formulated blockers were selectively toxic to A549 with minimal effects on BEAS-2B. Notably, a hormesis-like pattern (low-dose stimulation/high-dose inhibition in MTT) was evident for free Dof and Azi; encapsulation attenuated this effect for Dof but not for Azi. Co-administration with paclitaxel (Ptx) potentiated Dof-HPN cytotoxicity in A549 but did not enhance Azi-HPN, suggesting mechanism-dependent drug-drug interactions. Overall, HPN provides a biocompatible platform that improves K^+^ blocker delivery.

## 1. Introduction

Potassium (K^+^) channels are widely recognized as viable targets in cancer treatment due to their crucial roles in regulating cellular proliferation, apoptosis, migration, and volume homeostasis. Dysregulated expression of multiple K^+^ channel subtypes has been reported in various malignancies and is frequently associated with tumor aggressiveness and metastatic potential.

Kv10.1 (Eag1) is overexpressed in various tumor types but is infrequently present in healthy tissues, rendering it a highly selective therapeutic target. Kv1.3 and KCa3.1 have been implicated in cancer progression through their roles in regulating membrane potential, calcium signaling, and immune cell function within the tumor microenvironment. Inhibition of these channels has been reported to suppress tumor growth and modulate anti-tumor immune responses [1,2,3,4].

In contrast to traditional chemotherapies, potassium channel blockers may exhibit a higher therapeutic potential in cancer cells than in normal cells due to differential expression profiles. Numerous small-molecule inhibitors, monoclonal antibodies, and biologics, including nanobodies, have demonstrated effectiveness in preclinical models [5]. Although small-molecule inhibitors have been used to modulate ion channel function, their major limitation is the lack of specificity. This limitation often leads to off-target effects and dose-limiting toxicities, thereby restricting their clinical efficacy and safety [5,6,7]. Conversely, nano drug delivery systems (NDDS), tend to exhibit high target selectivity and reduced off-target interactions. Their diminutive size and adaptable structure may enable them to target cancer tumors through the Enhanced Permeability and Retention (EPR) effect, which is frequently unattainable by both small molecules and traditional antibodies [8]. This potential molecular precision is associated with fewer systemic adverse effects, particularly in non-target tissues, making nano drug delivery systems a potentially safer and more regulated therapeutic approach. Furthermore, biodegradable and biocompatible NDDS do not typically undergo metabolic transformation into hazardous byproducts and generally exhibit low immunogenicity [9]. NDDS may also serve as a promising alternative to small-molecule inhibitors for therapeutics targeting ion channels, especially where therapeutic accuracy and safety are critical. Consequently, inhibiting K^+^ channels may synergize with current treatments such as chemotherapy and immunotherapy, providing a multimodal approach to cancer therapy [5,6,7].

Nanogels are intramolecularly cross-linked, nano-sized polymeric networks generally produced via heterogeneous polymerization techniques. Composed of natural and/or synthetic polymers, these colloidal structures combine the properties of polymers and gels and can swell in suitable media. Nanogels are suitable for the controlled delivery of drugs, genes, peptides and enzymes [10,11].

2-Hydroxyethyl methacrylate (HEMA) is one of the monomers used in the synthesis of common polymeric nanomaterials. Its notable properties, such as low toxicity and tissue compatibility, have led to its use in numerous studies for biomedical applications [12,13,14]. Due to its biocompatibility, it has applications in drug delivery systems, artificial corneas, contact lenses [15], and as bone composite materials in tissue engineering. This natural monomer is a multipurpose carrier due to the presence of hydroxyl, carboxyl, and methyl groups in its structure [16,17,18].

Pectin, a natural polymer, is a heterogeneous complex polysaccharide of linear 1,4-linked α-D-galacturonic acid units. It is a hydrophilic polysaccharide found in higher plants, where it contributes to tissue rigidity and structural integrity. Due to its suitable physicochemical properties, it is widely used in the development of microgel systems and is commonly employed in environmental and biomedical fields [19,20,21,22].

Due to their environmental and unique chemical properties, pectin and HEMA monomers are of great importance in the design of multifunctional materials. In particular, considering the biocompatible structure of HEMA and its widespread use in drug delivery systems, together with the natural polymer pectin, it is possible to bind functional groups, such as drugs, to the particle surface and use them in drug delivery systems.

This study focused on the design and evaluation of cross-linked poly (HEMA)-pectin nanocomposites (HPN) as an innovative drug delivery system for potassium channel blockers in cancer treatment. HEMA and pectin were incorporated to manufacture nanogels proficient in encapsulating model blockers, dofetilide (Dof), and azimilide (Azi). The produced nanoparticles underwent comprehensive characterization, and their drug loading efficiency, release patterns, and cytotoxic effects were evaluated in both healthy bronchial epithelial cells (BEAS-2B) and lung cancer cells (A549).

Previously in a study conducted by Mena-Giraldo and colleagues [23], a photosensitizer polymeric nanocarrier was obtained by incorporating ultraviolet (UV) light-sensitive azobenzene molecules into chitosan. This resulted in the development of a UV-responsive carrier system. They evaluated the effect of UV light on the controlled release of drugs via nanobioconjugates. Nile red and dofetilide molecules were used as model release agents. They reported that dofetilide was released at a high rate within a short time after UV exposure. Our investigation involved the co-administration of Dof-HPN and Azi-HPN with the chemotherapeutic drug paclitaxel (Ptx) to both healthy and cancerous cells. This combination aimed to assess the efficacy of K^+^ channel blockers alone and in combination with a regular chemotherapy regimen and to study possible enhancement in the cytotoxic effectiveness of standard chemotherapy [24]. In this study, we sought to develop a safer and more effective strategy for targeted cancer therapy by integrating the unique advantages of polymer-based nanocarriers with the therapeutic potential of potassium channel inhibition. Cross-linked HEMA–pectin nanocomposites were engineered to encapsulate two model K^+^ channel blockers and modulate their release, cellular uptake, and cytotoxic profile. Because ion-channel modulators alone may be insufficient to induce robust anticancer effects, we further explored their combined use with paclitaxel as a representative chemotherapeutic agent. This approach aims to enhance therapeutic selectivity, reduce off-target toxicity, and provide a multimodal platform for future cancer treatments.

## 2. Results and Discussion

### 2.1. Characterization of HPN, Dof-HPN, and Azi-HPN

**Scheme 1:**
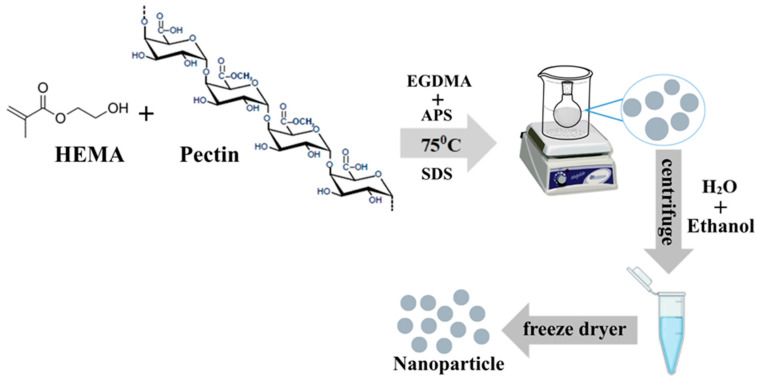
Schematic representation of the formation of nanogel particles from HEMA/pectin copolymers.

HEMA/Pectin copolymer was prepared using the surfactant SDS to form a surfactant-assisted aqueous nanogel polymerization system using Pectin and HEMA monomer. The polymerization reaction was initiated using APS as a redox initiator, and EGDMA was used as a crosslinker to link the polymer chains. The successfully synthesized HPN were dried using a lyophilizer. A schematic representation of the HPN synthesis is shown in Scheme 1.

### 2.2. FTIR Analysis of HPN

**Figure 1:**
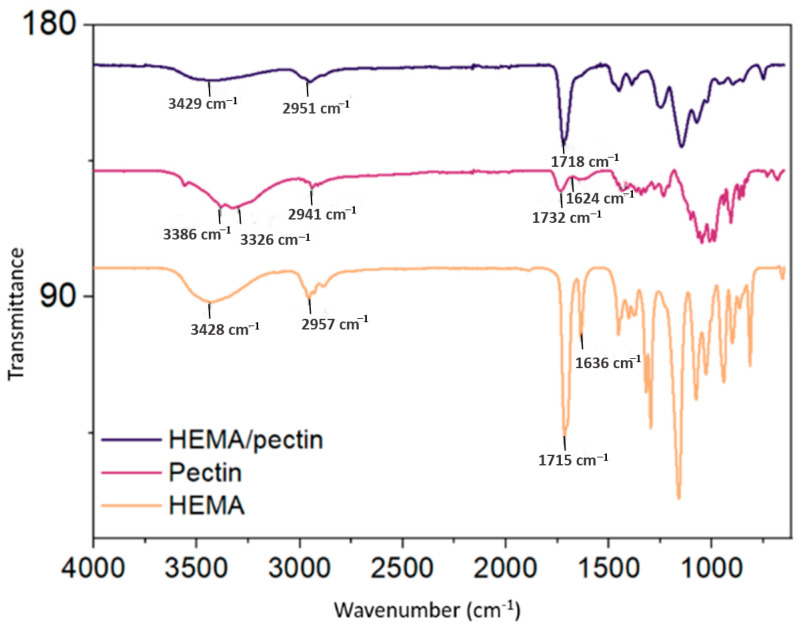
FTIR spectra of HEMA monomer, pectin, and HEMA/pectin (in the form of lyophilized NP).

Characteristic functional group peaks of HPN were analyzed by FTIR spectroscopy in the region of 650–4000 cm^−1^, as shown in Figure 1. They were compared with pectin and HEMA monomer to understand the structure of each polymer after synthesis of the HEMA/Pectin copolymer.

The FTIR spectrum for pure pectin shows broad bands of OH stretching at 3326 cm^−1^ and 3386 cm^−1^, while CH stretching is prominent at 2941 cm^−1^. Characteristic C=O stretching, belonging to carboxylic acid and ester carbonyl groups, was observed at 1624 cm^−1^ and 1732 cm^−1^, respectively. Furthermore, the fingerprint region, characterized by intense peaks in the 700–1200 cm^−1^ range, confirms the complexity of the polysaccharide structure [12,25].

The spectrum of the HEMA monomer contains the OH stretching band at 3428 cm^−1^ (a broad stretching band), and CH stretching is observed at 2957 cm^−1^. The C=O stretching observed at 1715 cm^−1^ is accompanied by stretching of the C=C double bond at 1636 cm^−1^. Similar data have been reported in the literature [3,26,27].

In the synthesized HPN system, the marked reduction and apparent overlap of the acidic C=O stretching of pectin at 1624 cm^−1^ (Figure 1) indicates that the acidic functional groups carried by the pectin chains are shielded within the NP structure by the synthesized HEMA chains. The C=O (ester carbonyl) stretching of pectin at 1732 cm^−1^ is markedly reduced and appears to be overlapped in the NP spectrum, while the C=O peak of HEMA at 1715 cm^−1^ shows a slight shift to 1718 cm^−1^, suggesting the copolymer interaction. The OH stretching observed at 3326 cm^−1^ and 3386 cm^−1^ of pectin disappeared from the spectrum, and instead, the OH stretching observed at 3428 cm^−1^ of HEMA was observed with a slight shift to 3429 cm^−1^ [28]. This suggests that the FTIR spectrum of the HPN shows strong similarity to the spectrum of the HEMA monomer, and that the pectin chains are integrated into the polymerized HEMA chain. Indeed, the decrease of the C=C stretching at 1636 cm^−1^ of HEMA supports that HEMA was successfully polymerized.

When the FT-IR results are evaluated as a whole, the presence of characteristic peaks from both components in the nanoparticle spectrum demonstrates the successful synthesis of the HPN structure. The pectin chains effectively interact with the HEMA chains to form a copolymer structure, and the HEMA is completely polymerized. Similar studies are also available in the literature review [29].

### 2.3. SEM and TEM Analysis of HPN

**Figure 2:**
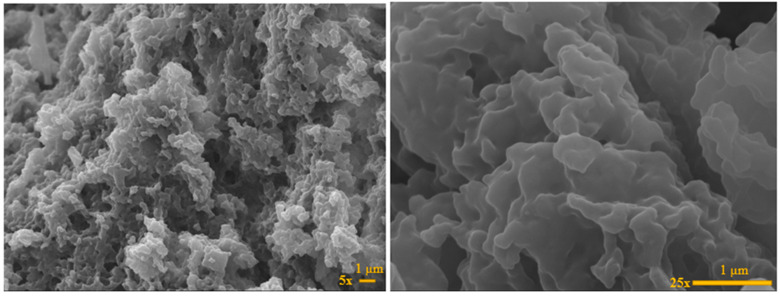
SEM images of HPN at different magnifications (5× left, and 25× right).

SEM studies were conducted on the synthesized HPN. Figure 2 shows SEM micrographs of the HPN at 5× and 25× magnifications. The images showed that the particles had a generally spherical morphology. Pectin and HEMA monomers are hydrophilic. SEM analysis was performed on the dried nanoparticles. However, due to their hydrophilic structure, the nanoparticles agglomerated, which may be associated with shrinkage effects occurring during the drying phase. In particular, the hydrophilic properties of the polymeric matrix and the evaporation of water molecules may contribute to the aggregation of the NPs and, consequently, the shrinkage behavior. The hydrophilic properties of 2-hydroxyethyl methacrylate (HEMA) and natural polysaccharide-derived pectin enable high water retention capacity in the synthesized HPN. Due to the hydrophilic nature of the HEMA and pectin components, the polymer matrix, initially swollen with a high concentration of water molecules, undergoes volumetric reduction and shrinkage as water is removed during the drying phase. In other words, the mechanical effects of shrinkage include strengthening of interchain interactions with water removal, and the polymer chains rearrange to form tighter and more compact structures [30].

**Figure 3:**
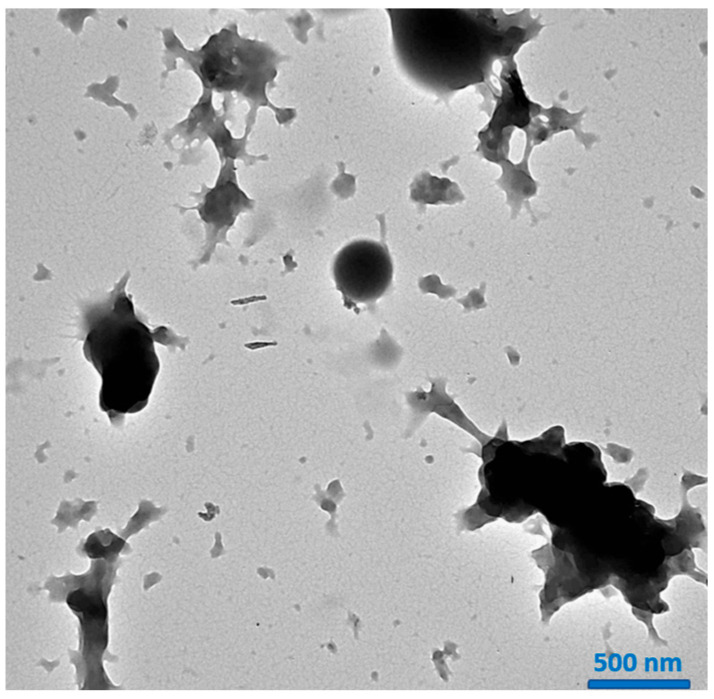
Representative TEM image of HPN illustrating morphological features.

The HPN were also imaged under a Transmission Electron Microscope (TEM) to investigate their morphology (Figure 3). The TEM images indicate that the nanogels exhibit a generally spherical morphology. Due to the highly swollen nature of the nanogels in aqueous media, TEM analysis was primarily employed for qualitative morphological evaluation rather than quantitative particle size determination.

### 2.4. Dynamic Light Scattering (DLS) Studies of HPN, Dof-HPN, and Azi-HPN

HPN was prepared as given in the specified ratios. Based on the dynamic light scattering and DLS data results shown in Table 1 and Supplementary Materials Figures S1–S5, empty nanoparticles with sizes of approximately 227–230 nm were obtained. The nanoparticle sizes determined by TEM analysis were also in close agreement with this value. These data indicate that the nanoparticles have a monodisperse distribution. Since the polydispersity index (PDI) value is ~0.15, it indicates that this system has a fairly homogeneous particle size distribution and that the synthesis process has been successfully optimized [31,32]. As can be seen in the graphs (Supplementary Materials S1–S5), no multimodal structure is observed in the analysis results according to the density and volume distributions. This indicates that the synthesis was carried out in a controlled manner. The single-peak distribution suggests a relatively uniform particle size distribution under the measured conditions. No significant particle agglomeration was detected in the DLS measurements performed after lyophilization and rehydration.

Encapsulation of Dof and Azi in HPN did not cause any change either in the size or in the polydispersity of particles. This evidence also indicates the successful encapsulation of K^+^ channel blockers in HPN particles.

### 2.5. X-Ray Diffraction (XRD) and Brunauer-Emmett Teller (BET) Analysis

X-ray diffraction (XRD) analysis was performed to investigate the structural charac-teristics of the HPN system. The corresponding XRD patterns are provided in the Supple-mentary Data S1, S6. The absence of sharp peaks typical of crystalline structure indicates that the copolymeric structure formed by cross-linking HEMA and pectin polymers has an amorphous character [33].

Pore volume distributions for the HPN were determined using Brunauer-Emmett Teller (BET) analysis. According to the analysis results, the surface area was 1.75 m^2^/g, the pore volume was 0.003 cm^3^/g, and the average pore size was 3.85 Ẫ (0.385 nm). The IUPAC classification indicated that the nanoparticles exhibited a microporous structure (pore diameter < 2 nm). This is an important parameter that directly affects the performance of nanoparticles [34]. The BET analysis performed on dried HPN samples revealed a microporous structure, reflecting the compact surface characteristics of the nanogels in the solid state. It should be noted that such gas-phase BET measurements do not represent the pore architecture under hydrated conditions, where HEMA–pectin nanogels undergo extensive swelling. Therefore, the BET-derived microporosity should be interpreted as an indicator of dry-state surface properties rather than a direct determinant of drug release behavior. Nevertheless, these results support the suitability of the nanogel surface for drug loading, while the release profiles in aqueous media are predominantly governed by polymer swelling, network relaxation, and drug–polymer interactions. It is noteworthy that gas-phase BET measurements on lyophilized hydrogels do not represent the actual pore architecture under hydrated (swollen) conditions, which is the state relevant for drug release. Hydrophilic HEMA/pectin networks are known to undergo substantial swelling in aqueous media, leading to transient expansion of polymer mesh size far beyond the Å-scale pores detected in the dried state.

### 2.6. EE% and DL% Capacity of HPN Against Dof and Azi

**Figure 4:**
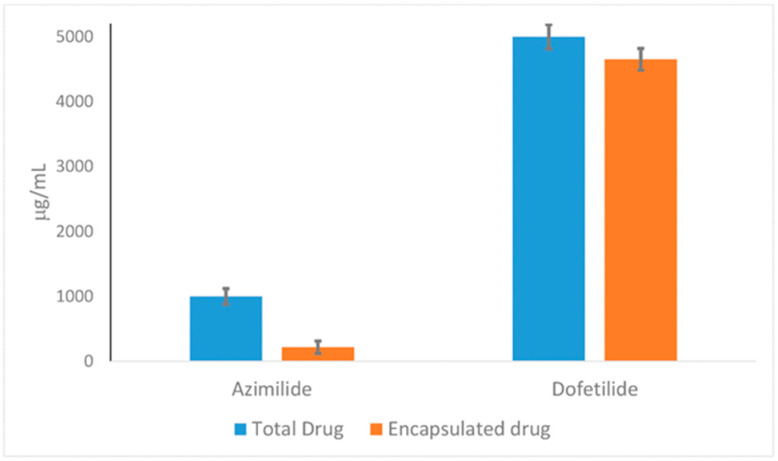
HPLC-determined total drug content and encapsulated drug amounts of azimilide- loaded HPN (Azi-HPN) and dofetilide- loaded HPN (Dof-HPN).

Encapsulation efficiency (EE%) and drug loading (DL%) quantify how effectively a nanoparticle formulation carries the active. EE% shows the fraction of the initial drug that ends up inside/associated with particles-indicating process efficiency, waste, and batch reproducibility. DL% indicates how much drug is contained per total nanoparticle mass-governing dose, injection volume, excipient burden, and payload-to-carrier ratio. Together, they guide optimization (polymer/lipid ratio, method, surfactant), predict release behavior, inform stability and scale-up, support cost and therapeutic index, and provide essential QC metrics for regulatory reporting [35]. The EE% and DL% of Dof and Azi in HPN were calculated using Equations (1) and (2). As shown in Figure 4, the amounts of total and encapsulated drugs were first quantified by HPLC and expressed as concentrations in µg/mL. These experimentally obtained values represent the primary quantitative data and were subsequently used to calculate encapsulation efficiency (EE%) and drug loading (DL%). Based on these calculations, EE% and DL% were determined as 93.01 ± 2.1% and 82.30 ± 3.1% for Dof and 21.72 ± 3.2% and 17.84 ± 2.9% for Azi, respectively.

### 2.7. Drug Release and Release Kinetic Studies of Dof-HPN and Azi-HPN

**Figure 5:**
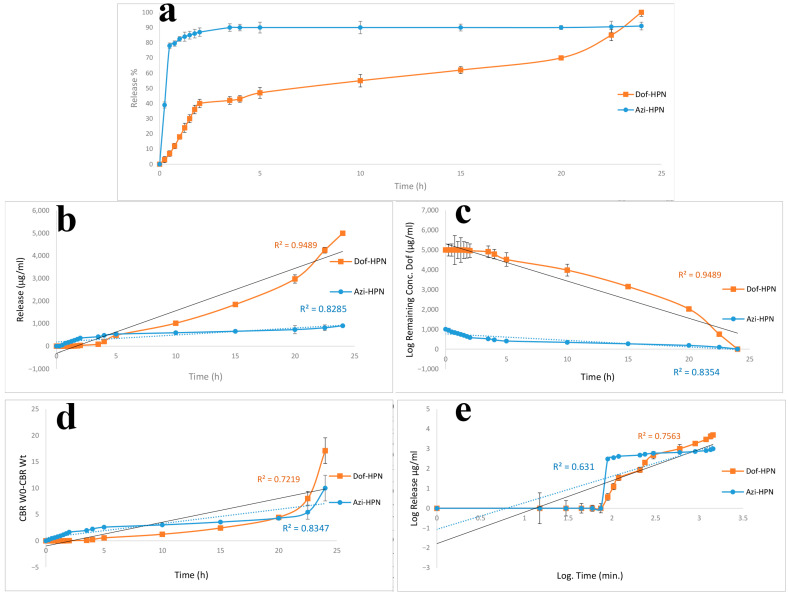
Drug release and release kinetic studies of Dof-HPN (orange) and Azi-HPN (blue), (**a**): %release, (**b**): Zero order, (**c**): First order, (**d**): Hixson-Crowell, and (**e**): Korsmeyer-Peppas mathematical models.

Understanding the release mechanism of drug delivery systems depends on various factors, such as polymer pore size and drug-carrier interactions. The affinity of the nanocarrier materials for the drug and the hydrophobic-hydrophilic nature of the loaded drug also significantly affect the release of the drug from the nanocarrier. Drug release was achieved in a mobile phase medium using HPN loaded with Dof and Azi, and Figure 5a shows their time-dependent release profile from HPN at 37 °C. The in vitro release profile of Dof-HPN, shown in Figure 5a, exhibits a characteristic release behavior consisting of two distinct phases. The first phase is the immediate release phase, observed during the first 3 h after application. This period is attributed to the rapid release of molecules located near the nanoparticle surface or with weak interactions. This rapid initial release is important for the rapid onset of therapeutic effects. The second phase, the sustained release phase, exhibits a slower, linear release profile. Approximately 80% of the drug was released within 20 h. 100% release was achieved within 24 h. In this phase, release depends primarily on drug diffusion through the nanoparticle matrix. This release ensures consistent drug delivery to the target site. The sustained phase following the initial burst release demonstrates the potential of HPN to provide both rapid therapeutic effects and long-term benefits. Long-term release is also thought to be related to the surface area and pore volume of the nanoparticles [36,37]. In Figure 5a, the release profile of Azi-HPN is also shown. This burst release profile from the HPN shows that drug release exceeds 80% within the first hour and reaches 90% within 2–3 h. This distinct and immediate release indicates the high concentration of the drug at or near the surface of the nanoparticle. This allows for rapid diffusion and rapid completion of the drug’s passage to the external environment. Immediate release profiles may be advantageous in clinical situations where a rapid onset of therapeutic effect is desired. However, this rapid release may also increase the risk of systemic toxicity [38].

The release mechanism of these two drugs from HPN was also investigated, and it was found that the release profile of Dof-HPN was best fitted to both zero and first-order kinetics with an R^2^ value of 0.9489. This indicates that the release follows a mixed process (burst + diffusion), which is in good agreement with the dual behavior observed in the release graph. In contrast, the release profile of Azi-HPN was best described by the first-order model, with an R^2^ value of 0.8354. Although this indicates a moderate correlation, it suggests that the model explains a substantial portion of the release behavior rather than providing a definitive mechanistic description. The relatively rapid release observed within the first 2–3 h may be attributed primarily to diffusion-driven processes. However, given the moderate goodness-of-fit, these findings should be interpreted cautiously.

### 2.8. In Vitro Cytotoxicity and Anticancer Efficacy of Dof-HPN and Azi-HPN

**Figure 6:**
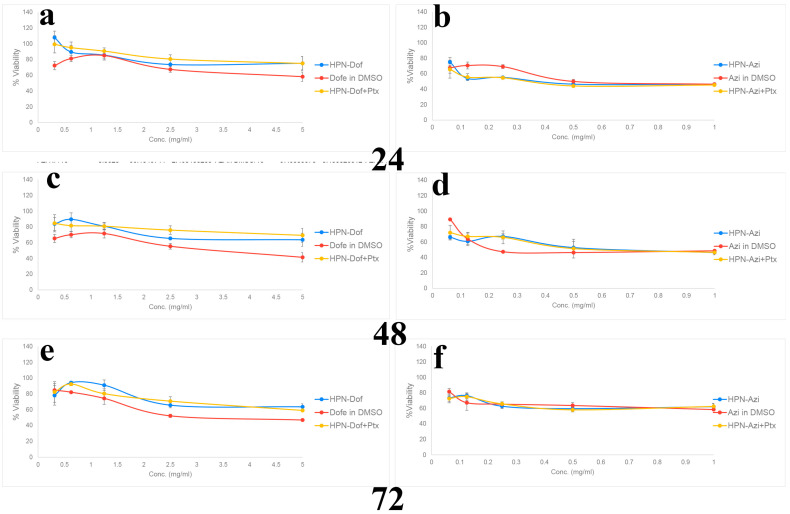
Cytotoxic effect of free and encapsulated Dof and its combination with Ptx (left column), and those of Azi-HPN (right column) on healthy human bronchial epithelial (BEAS-2B) cells at (**a**,**b**) 24 h. (**c**,**d**) 48 h, and (**e**,**f**) 72 h.

**Figure 7:**
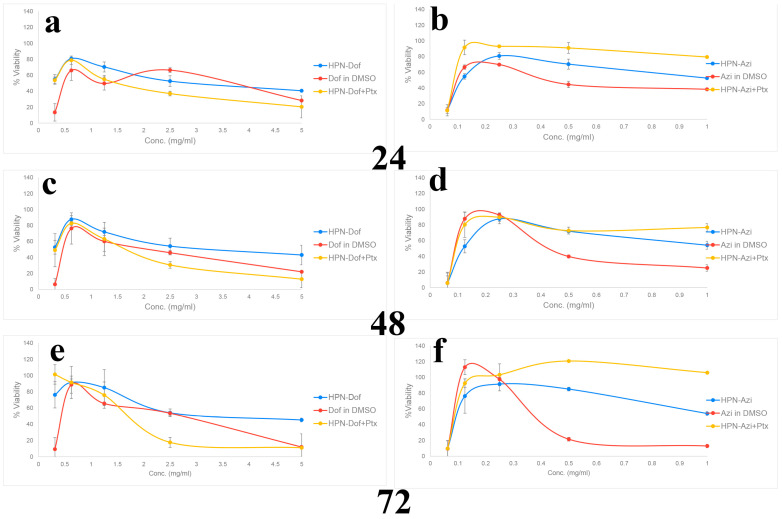
Cytotoxic effect of free and encapsulated Dof and its combination with Ptx (left column), and those of Azi-HPN (right column) on human lung adenocarcinoma (A549) cells at (**a**,**b**) 24 h. (**c**,**d**) 48 h, and (**e**,**f**) 72 h.

The MTT assay, which is based on mitochondrial metabolic activity, was used to determine the possible effects of drug-loaded nanoparticles on cellular viability. The therapeutic effects of HPN on the healthy human bronchial epithelial (BEAS-2B) and human lung adenocarcinoma (A549) cell lines after 24, 48, and 72 h of incubation are presented in Figure 6 and Figure 7, respectively.

As seen in Figure 6, the HPN system preserved the viability of healthy bronchial epithelial cells (cell viability 80% at the end of 72 h), suggesting that it may have a favorable safety profile in terms of biocompatibility. Both Dof and Azi showed a very mild increase in toxicity against healthy cells from 24 to 72 h, while free Dof was comparably more toxic than the encapsulated drug. It can be hypothesized that encapsulation leads to an apparent decrease in cyto-toxicity against healthy cells [39,40]. Co-administration of Ptx with drug-loaded HPN also did not change the low toxicity of nano-formulations.

As shown in Figure 7a,c,e, Dof-HPN showed a significant cytotoxic effect. Based on the results of different concentrations and 24–72 h of incubation, the decrease in cell viability observed supports the antiproliferative effect of Dof. However, free Dof and free Azi showed an irregular toxic behavior. These molecules showed a significant toxicity both at the highest and the lowest concentrations. This phenomenon is called hormesis [41]. Encapsulation of drug molecules in HPN eliminated the hormesis effect in the case of Dof-HPN. However, Azi-HPN showed the hormesis effect equal to free Azi, and the cytotoxicity of Azi-HPN in lower doses was much higher than its toxicity in higher doses.

Potassium channel blocker-loaded nanoparticles were administered to cells in vitro along with the chemotherapy agent paclitaxel to investigate the synergistic effects of the two agents. When the effects of Dof-HPN on cancer cells after 24–72 h of incubation were examined, Ptx in its IC50 value significantly increased the toxic effect of Dof-HPN on cell viability. However, Ptx didn’t show any synergistic effect with Azi.

**Figure 8:**
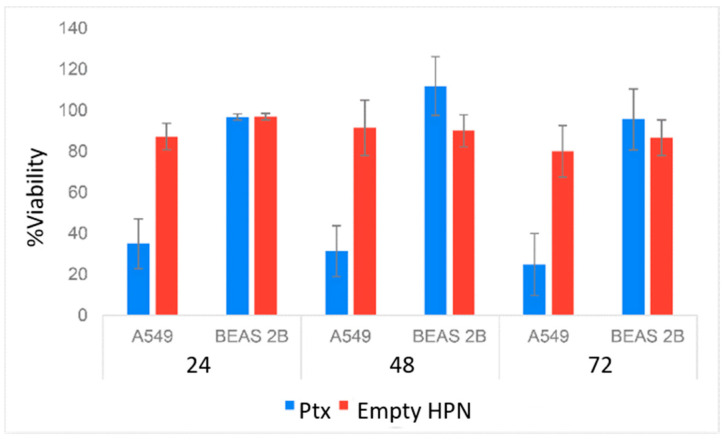
Cytotoxic activity of the control groups; empty HPN at its highest tested concentration (1 mg/mL) and free-Ptx at its IC50 value 7 nM.

Figure 8 shows the cytotoxic activity of the control groups used in the in vitro studies.

As it was experienced in the cytotoxicity assays of the experiment groups, Ptx did not show a significant activity on the healthy BEAS-2B cells, but it was toxic against cancer cells. Comparison of Figure 7 with Figure 8 shows that the toxicity of Ptx co-administered with the drug-loaded HPN was higher than free Ptx. The toxicity of free HPN was not significant on healthy cells nor on cancer cells.

In this study, HPN were synthesized and characterized using biocompatible HEMA and pectin, a natural polysaccharide. The structure of the synthesized polymer-based nanoparticles was extensively studied and validated using various characterization techniques, including TEM, SEM, FTIR, DLS, XRD, and BET. FTIR analysis confirmed the successful integration of HEMA into pectin.

The DLS, TEM, and SEM studies analyses demonstrated that HPN with an average particle size of approximately 230 nm are suitable for nanocarrier-based drug delivery systems [8]. The suitability of nanocarrier formulations for a particular drug administration route depends on their average diameter, PDI, and size stability, among other parameters. These parameters are key for the effective clinical application of nanocarrier formulations [42]. These physical properties of HPN show that these nanoparticles have a suitable structure for applications such as drug delivery systems and controlled drug release systems.

X-ray studies also revealed very important properties of the synthesized HPN. It was concluded that HPN possesses a microporous pore structure. Microporous pore structures have been reported in the literature in studies conducted with pectin or pectin-based composite nanoparticles. Hameed et al. [43] reported that the surface area of hydrogel particles prepared with pectin and chitosan was 2.261 m^2^/g, and the total pore volume was 0.0059 cm^3^/g. Compared to these data, the surface area of the synthesized HPN was smaller. These data suggest that the HEMA/pectin polymeric matrix has a more compact structure, allowing drug molecules to be retained within the nanoparticle matrix for longer periods. A small pore diameter may be advantageous for controlled release [44,45].

The delayed release of Dof from HPN confirmed this conclusion. Dof showed a higher EE% and DL% compared to Azi. It was also observed that the release period of Azi is extremely shorter than Dof, which indicates that Azi is incorporated with the shell part of the HPN rather than the core part. The mathematical models applied to the release profile of Dof-HPN and Azi-HPN also confirmed this inference. The release of Azi followed a first-order release (diffusion). In contrast, the release behavior of Dof-HPN was best fitted to both zero and first order, which shows burst release is also associated with diffusion [46].

Combining the release and X-ray studies reveals more details about the synthesized HPN particles. Azi showed rapid burst release (>80% within the first hour). This is consistent with its hydrophilicity, surface localization (as supported by low EE%), and the fact that upon hydration, the polymer network forms larger dynamic water channels that are not captured by BET measurements. These swollen-state pores facilitate fast aqueous diffusion. Dof exhibited sustained, biphasic release despite the same BET pore size. This behavior is governed not by dry-state physical pores but by drug–polymer interactions (hydrogen bonding, hydrophobic association) and its partial entrapment within the denser interior of the cross-linked HEMA–pectin matrix. Thus, the effective diffusional barrier is chemical–structural, not solely geometrical. Overall, the BET results confirm that HPN possesses a compact microporous structure when dried, while the hydrated polymer mesh is significantly more open, allowing drug diffusion governed by solvation, swelling, and drug–polymer affinity.

While the synthesized Dof-HPN and Azi-HPN were significantly toxic against A-549 cancer cells, their toxicity on the BEAS-2B cells was negligible. Studies in the literature have also reported that K+ blockers inhibit cell proliferation in cancer cells [3,35]. In this context, the observed cytotoxic effects may be partly associated with ion-channel–related mechanisms; however, direct evidence supporting such mechanisms was not obtained in the present study.

The very significant result obtained from in vitro cytotoxicity studies was the hormesis effect of potassium channel blockers on the cancer cells. Both free Dof and free Azi showed a hormesis effect. The cytotoxicity of the lowest concentration was as strong as the highest concentration, especially at the end of 72 h. This phenomenon, described similarly, has been reported in the literature [47,48].

In this context, the observed cytotoxic effects may be partly associated with ion-channel–related mechanisms; however, direct evidence supporting such mechanisms was not obtained in the present study. Encapsulation of Dof in HPN reduced the toxicity of the lowest concentration, which caused partial elimination of the hormesis effect at the end of 72 h. Encapsulation of Azi in HPN did not change its behavior in terms of hormesis. In case of the toxicity of Azi-HPN, the lowest concentration was the most toxic one, along with the lowest concentration of free Azi.

The highest ability of Azi in creating the hormesis effect may be attributed to its broader ion-channel profile and intracellular accumulation. Unlike Dof (a highly selective IKr blocker), Azi inhibits both IKr and IKs and, as a lipophilic weak base, can concentrate inside cells. Based on previous reports, in A549 cells such properties may be associated with membrane depolarization, increased L-type Ca^2+^ influx, and mitochondrial Ca^2+^ uptake, transiently enhancing ETC/NAD(P)H and inflating MTT (>100%) at low doses; however, these mechanisms were not directly investigated in the present study [49,50,51,52].

Co-administration of the chemotherapy agent Ptx with Dof-HPN enhanced its cytotoxic activity against cancer cells. However, this co-employment did not increase the cytotoxicity of Azi-HPN. It has been hypothesized that the antagonism of Azi at the chosen ratio/timing is possible. Alteration of membrane potential or ion currents by Azi may attenuate mitotic killing by Ptx; sequential rather than simultaneous administration may be more effective [53].

## 3. Conclusions

This study demonstrated the successful design and in vitro evaluation of cross-linked poly(HEMA)-pectin nanocomposites (HPN) as a polymer-based platform for the delivery of potassium channel blockers. By integrating the synthetic monomer HEMA with the natural polysaccharide pectin, a biocompatible hybrid composite was obtained that combined the tunable mechanical and chemical stability of synthetic polymers with the bioaffinity and degradability of natural polymers. The surfactant-assisted aqueous polymerization approach yielded nanogels with a relatively uniform particle size (~230 nm) and an amorphous, microporous morphology.

The HPN matrix effectively encapsulated the model blockers dofetilide (Dof) and azimilide (Azi), achieving high encapsulation efficiency and distinct release kinetics governed by polymer–drug interactions. Dof-HPN exhibited a biphasic zero/first-order release, ensuring prolonged therapeutic exposure, whereas Azi-HPN displayed rapid, diffusion-controlled release. Cytotoxicity studies revealed selective inhibition of A549 lung cancer cells with minimal toxicity toward normal bronchial cells, suggesting favorable biocompatibility under the tested conditions. A remarkable finding was the hormetic dose-response observed with the free drugs—attenuated upon encapsulation—highlighting the regulatory capacity of the polymer network in modulating drug–cell interactions. Furthermore, co-delivery with paclitaxel produced a synergistic effect for Dof-HPN, supporting the feasibility of combination strategies at the in vitro level.

Based on the literature survey conducted, studies involving the incorporation of dofetilide and azimilide into HEMA–pectin nanogel systems are very limited. In this context, the present study demonstrates the loading of these drugs into an HPN platform and evaluates their individual release behavior as well as their combination effects with paclitaxel. Overall, this work presents HPN nanogels as a versatile polymer-based system for investigating the encapsulation and release behavior of ion-channel modulators. The findings bridge the gap between polymer chemistry and oncology, underscoring the importance of polymeric composites not only as structural materials but also as functional therapeutic tools. Future studies will expand toward in vivo evaluation, mechanistic modeling of hormesis modulation, and customization of the HPN platform for other targeted therapies and bioactive molecules.

## 4. Materials and Methods

### 4.1. Materials

2-Hydroxyethyl methacrylate (HEMA, 97%) monomer, sodium dodecyl sulfate (SDS) used as a surfactant in the reaction medium, ethylene glycol dimethacrylate (EGDMA, 99%) as a crosslinker, and ammonium persulfate (APS, 98%) as a redox initiator were purchased from Sigma Aldrich (Darmstadt, Germany). Pectin C, a natural polymer obtained from citrus fruits with a degree of esterification ≥69%, was obtained from Carl Roth (Karlsruhe, Germany). Dofetilide (Dof) (≥98%) was purchased from Cayman (Ann Arbor, MI, USA), and Azimilide dihydrochloride (Azi) (≥97%) and Paclitaxel (semisynthetic, ≥98%) were purchased from Sigma-Aldrich (Darmstadt, Germany). All solutions were prepared using deionized water. The products used in the study were of analytical grade and were purchased from Sigma-Aldrich.

### 4.2. Methods

#### 4.2.1. Synthesis of HEMA/Pectin Nanoparticle (HPN)

The synthesis of cross-linked HEMA-pectin copolymeric nanoparticles (HPN) using HEMA monomer and pectin, was achieved by creating a surfactant-assisted aqueous nanogel polymerization system. The cross-linked synthesis was carried out in an oil bath under reflux and in a reaction flask at 75 °C. Pectin (5%, w/v) was obtained as a viscous solution in deionized water. 50 mL of 0.1 M surfactant SDS was used to form the surfactant-assisted aqueous nanogel polymerization system. For the synthesis, equimolar amounts of HEMA (154 mM) and pectin (154 mM) were mixed in an SDS solution until a homogeneous mixture was obtained. 10 mol% of the cross-linker EGDMA (ethylene glycol dimethacrylate), calculated based on the total molar amount of monomer, was added to the mixture. The reaction was then stirred until homogeneity was achieved. Then, 1% (based on the molar amount of total monomer) of the initiator APS (dissolved in 1 mL of water) was added to initiate the reaction and the pH of the reaction medium was 4.05. The reaction was allowed to take place for 6 h at a constant stirring speed (1000 rpm), and the resulting nanoparticles were first filtered through Whatman filter paper (>2.5 μm) to separate large particles. The filtrate was precipitated by centrifugation (6000 rpm). The precipitated nanoparticles were washed with 5 × 50 mL deionized water-ethanol (1:1) to remove unreacted monomer and crosslinker. After the washing process, they were dried with a lyophilizer (final yield: 68%) [54]. The lyophilized particles were kept at R.T. and were re-hydrated for drug loading experiments.

#### 4.2.2. Characterization of Nanoparticles (NPs)

To determine whether the monomers used contribute to the chemical structure of the synthesized HEMA/pectin nanoparticle, Fourier Transform Infrared spectroscopy (FTIR-ATR) (Perkin Elmer, Shelton, CT, USA, Spectrum 100) analyses were performed (ATR instrument, wavenumber range 650–4000 cm^−1^). Particle size distribution analyses of HEMA/pectin nanoparticles were measured on a Dynamic Light Scattering (DLS) instrument (Malvern Instruments Ltd., Malvern, UK). The dispersant was chosen as water, and the measurement angle was set as 173 with the refractive index of 1.59 and absorption of 0.010. All the measurements were repeated 3 times at RT. Particle size was reported as number, volume, and intensity distribution to ensure monodispersity of all particles. The surface morphology of the HEMA/Pectin nanoparticle was examined using a scanning electron microscope (SEM) JEOL JSM-7100-F model (JEOL, Tokyo, Japan). Transmission electron microscopy (TEM) analysis (JEOL JEM-1400 Plus) and X-ray diffraction (XRD) analysis (PANalytical Empyrean, λ = 1.54056 Å at 45 kV and 40 mA) were performed for HEMA/Pectin nanoparticles. Additionally, surface area measurements were determined by multipoint Brunauer–Emmett–Teller (surface area analysis (BET)) (Quantachrome QuadraSorb SI).

#### 4.2.3. Drug Loading to HPN

Dof and Azi, the K+ channel blockers, were uploaded to HPN (HPN-Dof and HPN-Azi). For this purpose, 1 mg of freeze-dried HPN was resuspended in 1 mL of ddH2O, and consecutive ultrasonication and vortexing were performed for 10 min. Since Azimilide dihydrochloride is water soluble, 1 mg of Azi was dissolved in 1 mL of water, and lyophilized HPN was re-hydrated in the Azi solution. However, Dof was not water-soluble, so 5 mg of Dof was separately dissolved in 1 mL of acetone and added to 1 mL of the rehydrated HPN. In both cases, consecutive ultrasonication and vortexing were repeated. Acetone was used as a temporary solvent for dofetilide loading due to its high volatility and complete miscibility with water. Following the loading process, acetone was removed by prolonged stirring under nitrogen, minimizing the risk of residual solvent. Although residual solvent quantification was not performed in this study, the applied processing steps and biological compatibility results indicate negligible solvent retention. The drug concentration was increased stepwise, and non-encapsulated drug molecules in aqueous media were detected as large aggregates, as detected by the DLS method. The drug amount was increased stepwise in 1 mg increments while keeping the nanoparticle amount and total volume constant. At each step, the formulation was mixed (vortexing/sonication) and analyzed by DLS. When the added drug exceeded the loading capacity of HPN, the excess drug remained outside the nanogels and formed insoluble/loosely associated aggregates, which appeared in DLS as the emergence of a secondary large-size population and/or a marked increase in apparent size and PDI. Therefore, the optimized drug concentration was defined as the highest concentration immediately below the first step at which aggregation was detected by DLS, ensuring a monodisperse formulation suitable for subsequent experiments.

Loaded drug concentrations of HPN-Dof and HPN-Azi were determined using HPLC.

#### 4.2.4. Quantitative Measurement of Dof and Azi Using HPLC and Method Validation

A new liquid chromatographic method was developed for the simultaneous determination of Dof and Azi using a single chromatographic system, with slight adjustments in the mobile phase composition to ensure optimal resolution for each analyte. The HPLC device (Shimadzu LC-20A system, Kyoto, Japan) was equipped with an autosampler, a column oven compartment, and a DAD detector. Separation was performed using a cyano column (250 × 4.6 mm, 5 µm i.d.) with a flow rate of 1.0 mL/min and the column oven temperature of 40.0 °C. Dof was detected at 228 nm, whereas Azi was detected at 343 nm. An isocratic mobile phase system consisting of acetonitrile, methanol, and 10 mM o-phosphoric acid containing 0.1% trimethylamine was used. The mobile phase composition was 10:10:80 (v/v/v) for Dof, and 25:25:50 (v/v/v) for Azi. For sample preparation, 100 µL of the sample was taken and diluted to 1 mL with the mobile phase, followed by vortexing for 30 s. Finally, 25 µL was injected into the HPLC system. Chromatographic data acquisition, analysis, and reporting were performed using the LC-Solution system software (LCsolution Multi LC/PDA 1.25, Shimadzu Corporation, Kyoto, Japan). Under these conditions, the retention times of Azi and Dof were 7.5 min and 9.7 min, respectively.

The developed method was validated using the International Conference on “Harmonized Tripartite Guideline Validation of analytical procedures” [55]. System suitability was evaluated by six replicate injections of the standard solution corresponding to the 100% level (50 µg/mL for both Azi and Dof), and the %RSD values for peak area and retention time were found to be less than 2%, confirming acceptable system performance. To assess the method selectivity, the system was injected with the mobile phase, standard solutions of Azi and Dof, the HPN formulation, and placebo solutions, and no interfering peaks were observed at the retention times of either Azi or Dof, indicating good method selectivity. Carryover was evaluated by performing blank injections (mobile phase) immediately after the highest concentration sample injections, and no peaks corresponding to Azi or Dof were observed. Linearity was evaluated over the range of 5.0–100.0 µg/mL using six calibration points (5.0, 12.5, 25.0, 50.0, 75.0, and 100.0 µg/mL) for both Azi and Dof, with mean correlation coefficients of ≥0.99. In accordance with the study parameters, LOD values were found to be 0.39 and 0.08 µg/mL, and LOQ values were found 1.28 and 0.26 µg/mL for Azi and Dof, respectively. Assay recovery and precision of Azi and Dof from HPN at 5.0, 50.0, and 100.0 µg/mL were evaluated. The mean recoveries for the two active pharmaceutical ingredients (APIs) loaded to HJPN were calculated as 95.2–99.7%. Precision was evaluated in terms of both intra-day and inter-day repeatability. The RSD% values for intra-day repeatability ranged from 0.015% to 1.274%, while those for inter-day repeatability ranged from 0.102% to 0.529%. These results comply with the commonly accepted criterion that RSD% values which is determined as less than 2.0%. The validated method was successfully applied to the quantification of two APIs in HPN and during the drug release studies.

#### 4.2.5. Drug Release and Release Kinetic Studies of Dof-HPN and Azi-HPN

Dof and Azi show affinity to membrane materials. Therefore, a modified method was used to study the release behavior. The optimized formulations were subjected to dialysis using an AmiconVR Ultra Centrifuge 15 mL tube (MWCO 3 kDa). A sample from the formulation was retained to quantify the concentrations of Dof and Azi at the baseline, designated as 100%. Precisely quantified volumes of 70% ethanol were placed in the basal compartment of the dialysis tube, which remains isolated from the apical sample. The dialysis tube was placed in a shaker incubator at 37 °C and centrifuged at 6000 rpm for 10 min at specified time intervals. The concentrations of Dof and Azi in basal medium post-centrifugation were quantified using the HPLC method as previously outlined, and a corresponding percentage was calculated based on the initial amounts of Dof and Azi at time zero. Fresh water was added to both apical and basal media to maintain constant total volumes. The sample and ethanol never came into contact with each other, and the release occurred only in the aqueous medium of the sample itself. At the end of the dialysis experiment, a sample from the apical medium was collected to verify the precision of the release data. The kinetics of Dof and Azi release from the improved HPN were analyzed using zero-order, first-order, Hixson-Crowell, and Korsmeyer–Peppas kinetic models. The R2 values for each trendline were computed, and the model exhibiting the highest R2 value was selected as the optimal fit [56]. Although the Korsmeyer-Peppas model is most rigorously applied to the first 60% of cumulative release [57], in the case of Azi-HPN, the release occurred extremely rapidly, with almost the entire drug content being released within the first hour. As a result, restricting the fit to only the initial 60% would correspond to a very narrow and statistically insufficient portion of the dataset, leading to an unreliable model fit. For this reason, and as commonly done for burst-release systems in the literature, we applied the model to the full release profile to obtain a more meaningful kinetic interpretation.

#### 4.2.6. Encapsulation Efficiency (EE%) and Drug Loading (DL%) Capacity

It is hypothesized that Dof and Azi are dissolved in the aqueous media in amounts equal to their water solubility, and the excess amount of Dof and Azi is encapsulated in HPN. So, Dof-HPN and Azi-HPN were centrifuged at 6000 rpm for 10 min to separate the Dof and Azi molecules in the aqueous media. The Dof and Azi concentrations in the samples obtained from supernatants were calculated by direct injection to HPLC using the above-mentioned method. The weight of the encapsulated drug was calculated by subtracting the amount of Dof and Azi in the supernatant from their total amounts in the formulation. EE% was calculated according to Equation (1).

To calculate the drug loading capacity, the total amounts of drug in the optimized formulations and the total weight of HPN were considered to calculate DL% according to Equation (2) [24].

(1) $$Encapsulation\;Efficiency\;\%=\frac{Weight\;of\;total\;drug-Weight\;of\;free\;drug}{Weight\;of\;total\;drug\;used\;in\;formulation}\times 100$$

(2) $$Drug\;Loading\;Capacity\;\%=\frac{Weight\;of\;total\;drug-Weight\;of\;free\;drug}{Weight\;of\;HPN}\times 100$$

#### 4.2.7. In Vitro Cytotoxicity and Anticancer Efficacy of Dof-HPN and Azi-HPN

Human bronchial epithelial cell lines (BEAS-2B) were obtained from European Collection of Authenticated Cell Cultures (ECACC), and human lung adenocarcinoma cell lines (A549, CCL-185) from American Type Culture Collection (ATCC). Cells were grown under standard cell culture conditions in Dulbecco’s Modified Eagle Medium: Nutrient Mixture F-12 (DMEM-F12; Gibco, MD, USA) containing 10% fetal bovine serum (FBS; Gibco, Baltimore, MD, USA) and 1% penicillin/streptomycin. Cells were cultured at 37 °C in a humidified incubator with 5% CO_2_.

To assess the potential cytotoxic effects of Dof-HPN and Azi-HPN on healthy (BEAS-2B) and tumor (A549) cells MTT (3-(4,5-dimethylthiazol-2-yl)-2,5-diphenyltetrazolium bromide) assay was utilized. Both cell lines were distributed into 96-well plates containing 1 × 10^4^ cells per well and incubated overnight to allow cells to adhere to the surface. Subsequently, they were treated with drug-loaded HPN at different concentrations (6.25, 12.5, 25, 50, and 100 μg/mL) for 24, 48, and 72 h. For drug-loaded systems (HPN–Dof and HPN–Azi), the nanogel concentration was kept constant, and the corresponding effective drug dose was calculated based on the experimentally determined drug loading efficiency. Thus, comparisons between empty HPN and drug-loaded HPN were normalized to the same nanogel mass, while biological effects of the loaded formulations reflect the actual amount of drug released from a defined quantity of nanogel. Free HPN, Dof, and Azi were applied at the equivalent concentrations.

Ptx was applied at a constant concentration of 7 nM, which is reported as its IC50 value [58].

After treatment, the media was collected, fresh media was applied, and MTT (final concentration 0.1 mg/mL) solution was applied to the cells and incubated at 37 °C for 4 h. The MTT assay measures cell viability based on mitochondrial metabolic activity. Viable cells contain active dehydrogenase enzymes that reduce the yellow tetrazolium salt (MTT) to insoluble purple formazan crystals, whose quantity, quantified spectrophotometrically, correlates directly with the number of living cells [59]. After removing the supernatant, Dimethyl sulfoxide (DMSO) was added to each well to dissolve formazan crystals. The mixture was incubated at room temperature in the dark for 30 min. Absorbance measurements were recorded using a microplate reader at 570 nm wavelength (BioTek Instruments, Inc., Winooski, VT, USA). The percentage of viability fractions in the concentration fraction was calculated relative to the control group. All experiments were performed in triplicate, and the results are presented as the mean ± standard deviation.

Statistical analysis was performed using one-way analysis of variance (ANOVA) to evaluate differences among experimental groups. When a statistically significant difference was detected, Tukey’s honestly significant difference (HSD) post-hoc test was applied to determine pairwise significance within the groups. This statistical approach was used for the analysis of cell viability (MTT) data and comparative in vitro treatment groups, and results were considered statistically significant at *p* < 0.05.

## Figures and Tables

**Table 1:** Size Distribution and PDI of HPN, Dof-HPN, and Azi-HPN.

Size Distribution	Number	Volume	Intensity	PDI
HPN	220.0 ± 17.36	230.7 ± 22.76	227.9 ± 24.98	0.152
Dof-HPN	230.3 ± 19.34	240.1 ± 23.85	235.1 ± 25.01	0.181
Azi-HPN	228.0 ± 16.83	238.3 ± 20.55	232.1 ± 22.08	0.130

## Data Availability

The data supporting this study—including raw and processed characterization files (FTIR, DLS, TEM/SEM, XRD, BET), drug loading/encapsulation spreadsheets, release profiles, and cytotoxicity/readout tables with analysis scripts—are available from the corresponding author on reasonable request. Data are not publicly archived at this time due to institutional intellectual-property considerations. All requests will be evaluated for legitimate research purposes.

## Supplementary Materials

The following supporting information can be downloaded at: https://www.mdpi.com/article/10.3390/gels12010013/s1. Figure S1: Size of HPN by Intensity distribution. Figure S2: Size of HPN by Number distribution. Figure S3: Size of HPN by Volume distribution. Figure S4: Size of Azi-HPN by Number distribution. Figure S5: Size of Dof-HPN by Number distribution. Figure S6: XRD analysis image of HPN. Figure S7: Calibration curve of dofetilide obtained by HPLC analysis. Figure S8: Calibration curve of azimilide obtained by HPLC analysis. Figure S9: Representative HPLC chromatogram of dofetilide. Figure S10: Representative HPLC chromatogram of azimilide.

## Abbreviations

The following abbreviations are used in this manuscript:

A549	Human lung adenocarcinoma cell line
Azi	Azimilide
BEAS-2B	Human bronchial epithelial cell line
BET	Brunauer–Emmett–Teller (surface area analysis)
DLS	Dynamic Light Scattering
DL%	Drug Loading (percentage)
DMSO	Dimethyl sulfoxide
Dof	Dofetilide
EPR	Enhanced Permeability and Retention (effect)
EE%	Encapsulation Efficiency (percentage)
FTIR	Fourier Transform Infrared Spectroscopy
HEMA	2-Hydroxyethyl methacrylate
HPN	HEMA–Pectin Nanoparticles
hERG/IKr	Human ether-à-go-go–related gene/rapid delayed rectifier K^+^ current
IKs	Slow delayed rectifier K^+^ current
K^+^	Potassium ion
MTT	3-(4,5-Dimethylthiazol-2-yl)-2,5-Diphenyltetrazolium Bromide assay
NP	Nanoparticle
PDI	Polydispersity Index
Ptx	Paclitaxel
SEM	Scanning Electron Microscopy
TEM	Transmission Electron Microscopy
