# Supplementary material for: Design and In Vitro Evaluation of Cross-Linked Poly(HEMA)-Pectin Nano-Composites for Targeted Delivery of Potassium Channel Blockers in Cancer Therapy
Source: Gels. 2025 Dec 24;12(1):13. doi: 10.3390/gels12010013 (PMC12841014; doi:10.3390/gels12010013)
Supplement: Supplementary file 1 [file gels-12-00013-s001.zip › gels-4030570-supplementary.pdf]

Design and In Vitro Evaluation of Cross-Linked Poly(HEMA)-Pectin Nano-Composites for Targeted Delivery of Potassium Channel Blockers in Cancer Therapy

Gizem Ozkurnaz Civir, Fatemeh Bahadori, Ozgur Ozay, Gamze Ergin Kizilçay, Seyma Atesoglu, Burak Celik

Supplementary Materials

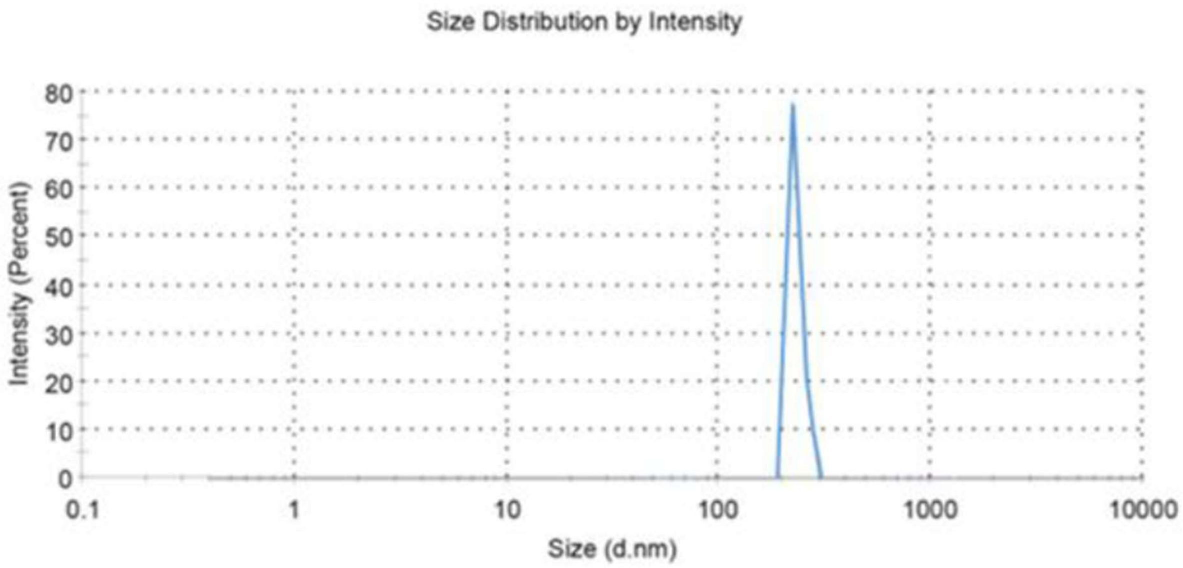

Figure S1: Size of HPN by Intensity distribution.

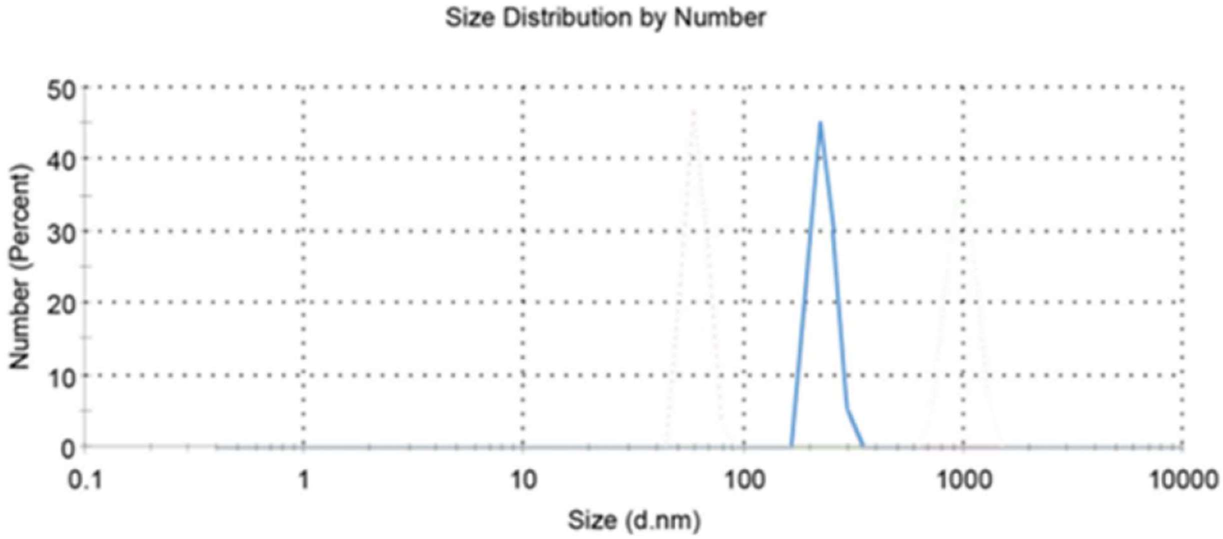

Figure S2: Size of HPN by Number distribution.

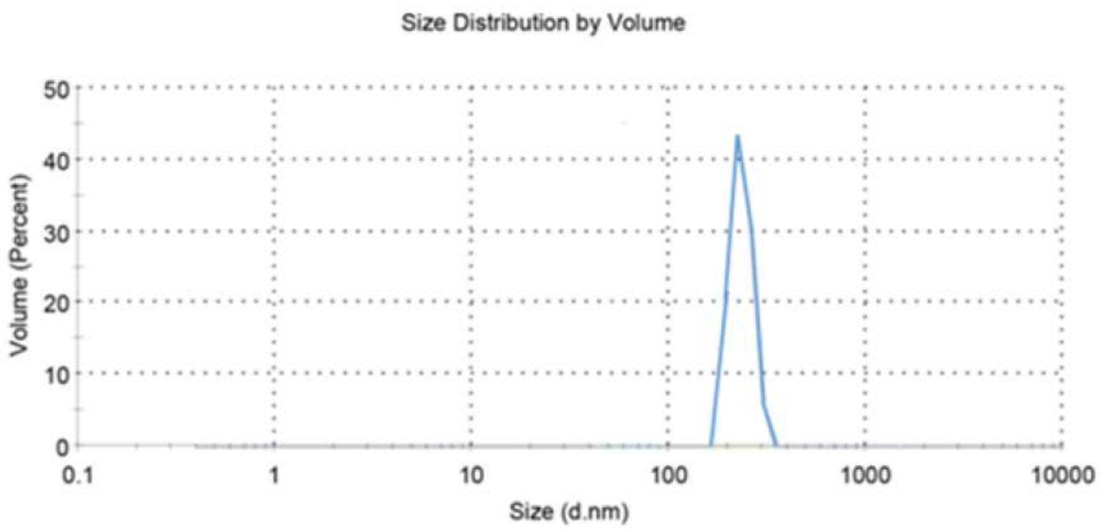

Figure S3: Size of HPN by Volume distribution.

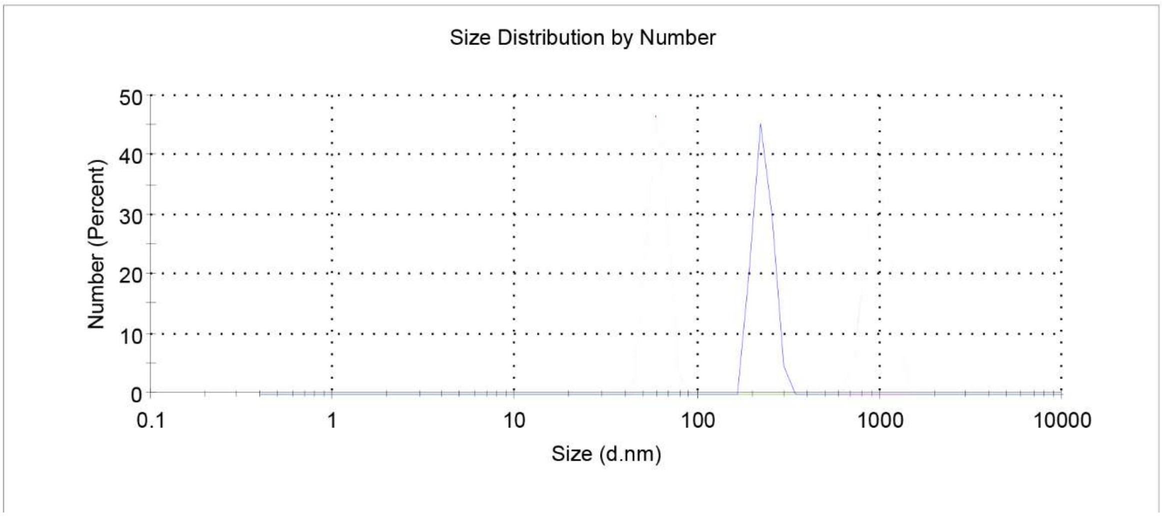

Figure S4: Size of Azi-HPN by Number distribution.

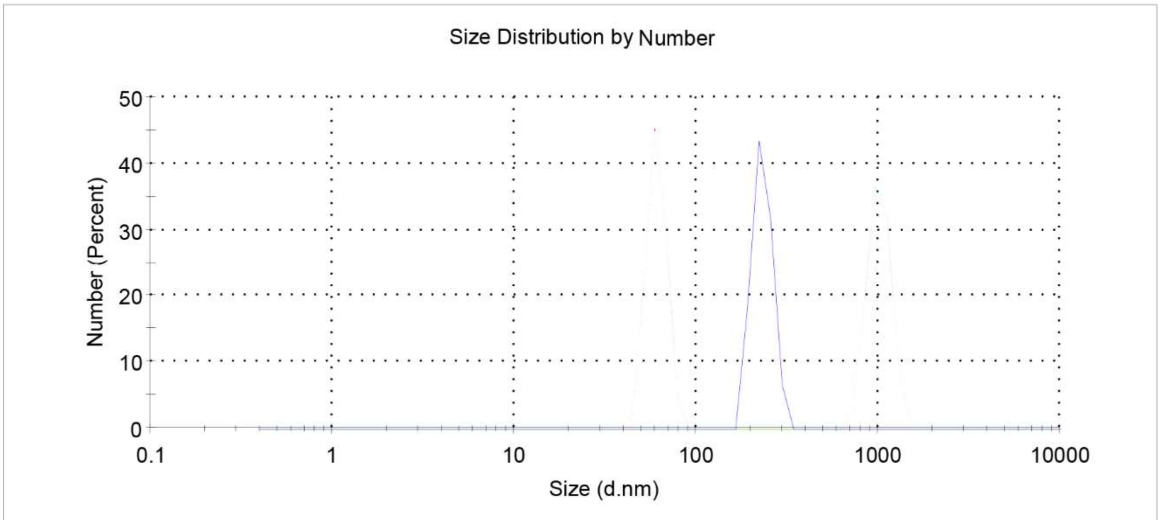

Figure S5: Size of Dof-HPN by Number distribution.

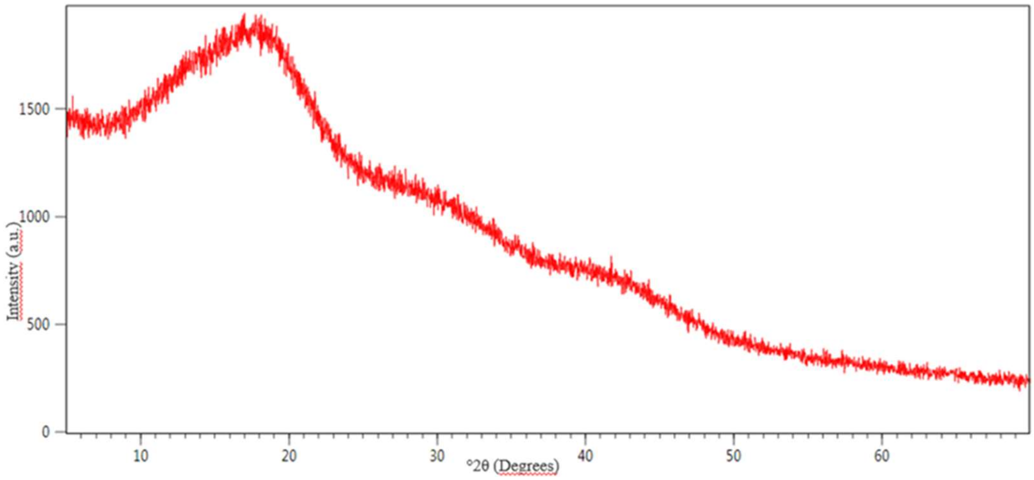

Figure S6: XRD analysis image of HPN.

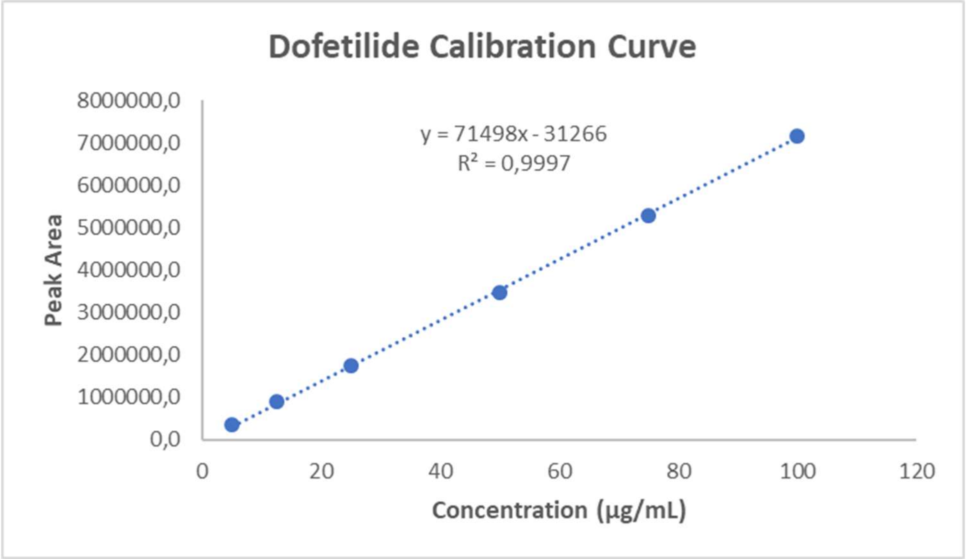

Figure S7: Calibration curve of dofetilide obtained by HPLC analysis.

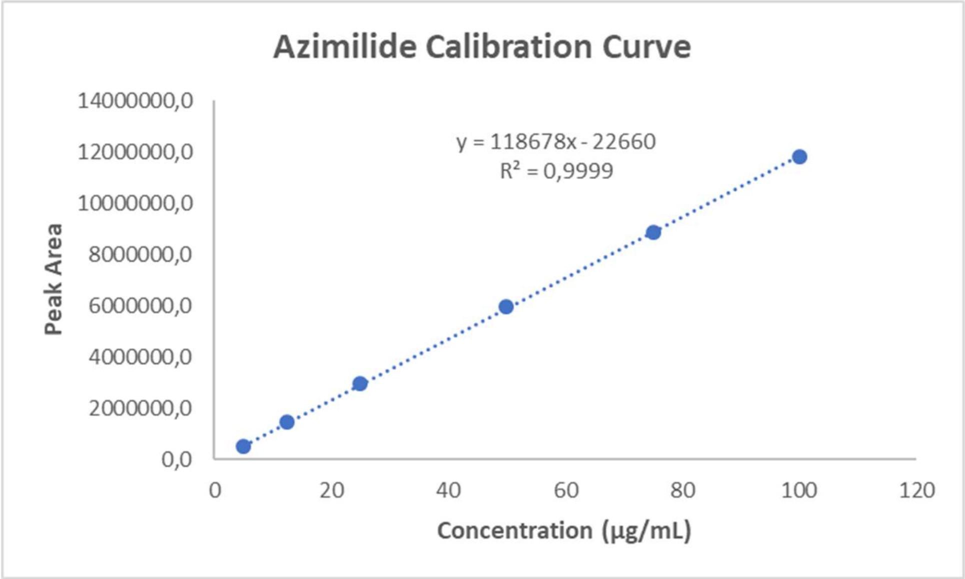

Figure S8: Calibration curve of azimilide obtained by HPLC analysis.

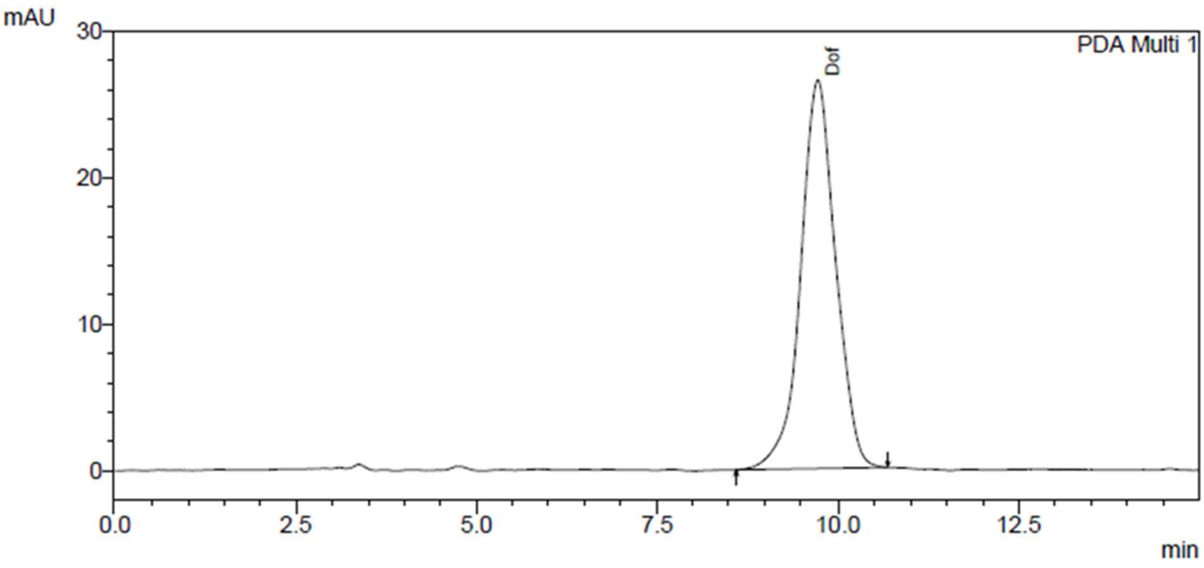

Figure S9: Representative HPLC chromatogram of dofetilide.

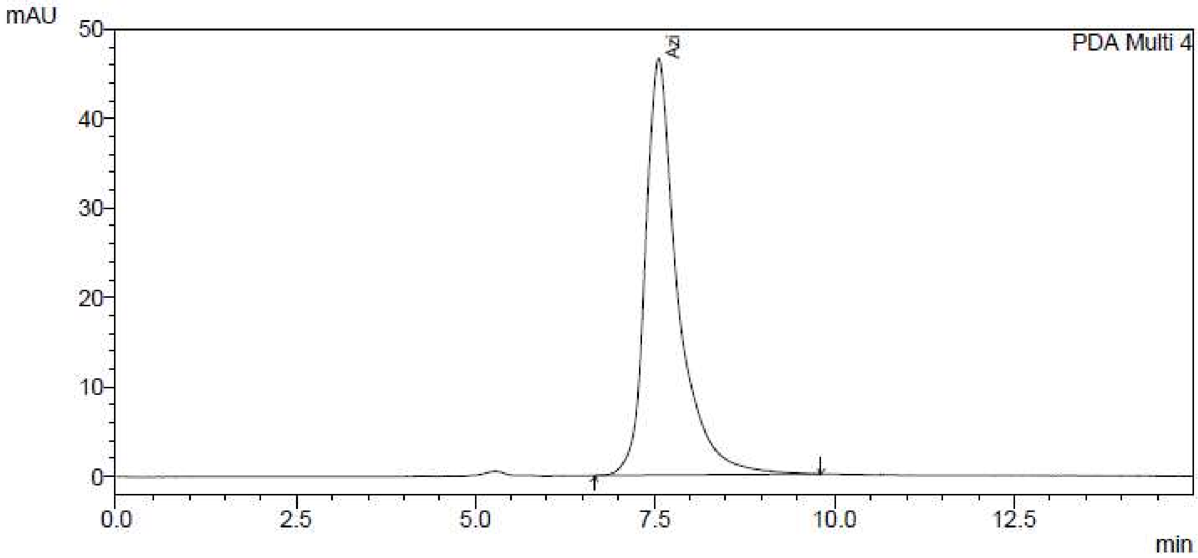

Figure S10: Representative HPLC chromatogram of azimilide.
